# Supplementary material for: Cloud BioLinux: pre-configured and on-demand bioinformatics computing for the genomics community
Source: BMC Bioinformatics. 2012 Mar 19;13:42. doi: 10.1186/1471-2105-13-42 (PMC3372431; doi:10.1186/1471-2105-13-42)
Supplement: Additional file 1 — Supplementary 1 Cloud BioLinux software documentation in the form of a mini, self-contained website. Users need to download and uncompress the .zip file, and open through a web browser the "index.html" file available on the main directory. (ZIP 1823 kb). [file 1471-2105-13-42-S1.ZIP › Cloud-BioLinux-Package-Documentation/docs/tree-puzzle.html]

Bio-Linux Software Documentation Pages

Back to search form

## tree-puzzle

|  |  |
| --- | --- |
| Name | tree-puzzle |
| Description | From the tree-puzzle website:  **TREE-PUZZLE** is a computer program to reconstruct phylogenetic trees from molecular sequence data by maximum likelihood. It implements a fast tree search algorithm, quartet puzzling, that allows analysis of large data sets and automatically assigns estimations of support to each internal branch.  **TREE-PUZZLE** also computes pairwise maximum likelihood distances as well as branch lengths for user specified trees. Branch lengths can be calculated with and without the molecular-clock assumption. In addition, **TREE-PUZZLE** covers likelihood mapping, a method to investigate the support of a hypothesized internal branch without computing an overall tree and to visualize the phylogenetic content of a sequence alignment.  **TREE-PUZZLE** also conducts a number of statistical tests on the data set (chi-square test for homogeneity of base composition, likelihood ratio to test the clock hypothesis, one and two-sided Kishino-Hasegawa test, Shimodaira-Hasegawa test, Expected Likelihood Weights). The models of substitution provided by TREE-PUZZLE are GTR, TN, HKY, F84, SH for nucleotides, Dayhoff, JTT, mtREV24, BLOSUM 62, VT, WAG for amino acids, and F81 for two-state data.  Rate heterogeneity is modeled by a discrete Gamma distribution and by allowing invariable sites. The corresponding parameters (except for GTR) can be inferred from the data set.  **References:**  Adachi, J., and M. Hasegawa. 1996. MOLPHY: programs for molecular phylogenetics, version 2.3. Institute of Statistical Mathematics, Tokyo.    Adachi, J., and M. Hasegawa. 1996. Model of amino acid substitution in proteins encoded by mitochondrial DNA. *J. Mol. Evol.* 42: 459-468. [Entrez]    Dayhoff, M. O., R. M. Schwartz, and B. C. Orcutt. 1978. A model of evolutionary change in proteins. In: Dayhoff, M. O. (ed.) Atlas of Protein Sequence Structure, Vol. 5, Suppl. 3. National Biomedical Research Foundation, Washington DC, pp. 345-352. |
| Homepage | http://www.tree-puzzle.de/ |
| Remote Documentation | http://www.tree-puzzle.de/tree-puzzle.pdf |
